# A novel fractional-order dead-time compensating controller for the wireless networks

**DOI:** 10.1038/s41598-023-44515-7

**Published:** 2023-10-17

**Authors:** P. Arun Mozhi Devan, Rosdiazli Ibrahim, Madiah Omar, Kishore Bingi, M. Nagarajapandian, Hakim Abdulrab

**Affiliations:** 1 Department of Electrical and Electronic Engineering, Universiti Teknologi PETRONAS, Seri Iskandar, 32610 Malaysia; 2 Department of Chemical Engineering, Universiti Teknologi PETRONAS, Seri Iskandar, 32610 Malaysia; 3 Department of Electronics and Instrumentation Engineering, Sri Ramakrishna Engineering College, Coimbatore, 641022 Tamil Nadu India

**Keywords:** Engineering, Electrical and electronic engineering

## Abstract

Wireless technology is becoming increasingly critical in industrial environments in recent years, and the popular wireless standards are WirelessHART, ZigBee, WLAN and ISA100.11a, commonly used in closed-loop systems. However, wireless networks in closed-loop control experience packet loss or drops, system delay and data threats, leading to process instability and catastrophic system failure. To prevent such issues, it is necessary to implement dead-time compensation control. Traditional techniques like model predictive and predictive PI controllers are frequently employed. However, these methods’ performance is sluggish in wireless networks, with processes having long dead times and set-point variations, potentially affecting network and process performance. Therefore, this paper proposes a fractional calculus-based predictive PI compensator for wired and wireless networks in the process control industries. The proposed technique has been simulated and evaluated on industrial process models, including pressure, flow, and temperature, where measurement and control are carried out wirelessly. The wireless network’s performance has been evaluated based on packet loss, reduced throughput, and increased system latency. The proposed compensator outperformed traditional methods, demonstrating superior set-point tracking, disturbance rejection, and delay compensation characteristics in the performance evaluations of the first, second, and third-order systems. Overall, the findings indicate that the proposed compensator enhances wireless networks’ performance in the process control industry and improves system stability and reliability by reducing almost half of the overshoot and settling an average of 8.3927% faster than the conventional techniques in most of the systems.

## Introduction

Networked control systems (NCS) have played a crucial role in industrial process control for many years^1–3^. With the rapid advancement of technology, significant improvements have been made in this area. Advancements in technology involve^4,5^

- the shift from wired to wireless technology,
- the use of digitalized instruments in place of analog-based ones, and
- the adoption of auto-diagnostic intelligent instruments instead of manually analyzed digital equipment.

These developments have greatly enhanced the efficiency and effectiveness of industrial processes.The advancements in communication technology have brought about a significant revolution in control strategies over the years^6,7^. The evolution of this technology has been evident, with the single electronic control loop of the 1960s transitioning to a single-loop digital controller in the 1970s, followed by a multi-loop digital controller for individual process plants in the 1980s^8,9^. The evolution of control systems has led to the development of wireless digital controllers, which are currently at the forefront of this technological advancement. The state-of-the-art communication protocols have dramatically impacted industrial plants, which traditionally rely on wired communication protocols to connect controllers and other plant components^10,11^. However, wired networked control systems have limitations in scalability, distribution, self-organizing capabilities, and dynamic topology, which are critical for the efficient operation of modern industrial processes. As such, adopting wireless digital controllers represents a significant step forward in the industry and is expected to improve overall performance and productivity^12,13^.

The development of wireless communication is prompted by the inadequacies of wired NCS, which include high cost, lengthy installation time, and the need for a significant number of cables for maintenance purposes^14,15^. Wireless communication is attractive during resilience, self-configured and quicker installation is required^16,17^. However, some wireless communication protocols, such as Bluetooth, WLAN, and Wi-Fi, are only suitable for home and office use due to the high level of reliability, accuracy, timeliness, and losslessness required in industrial applications^18,19^. ZigBee, WirelessHART, and ISA100.11a Wireless were introduced for industrial applications to meet these requirements^20,21^. Despite their ability to meet these requirements, these standards are limited to monitoring-based applications, with few successful attempts made to control applications^2^.

## Related works

Terry et al.^22^ have conducted initial research on wireless control. They proposed PID control methods for implementing wireless control and presented the performance to demonstrate the significance of the wireless actuators. The tests involved introducing set-point changes and unmeasured load disturbances, and the results showed that stable control is observed for these conditions using the wireless process control valve. In the same way, many researchers also discussed the challenges faced in implementing wireless control applications, particularly regarding feedback latency and battery longevity^23–26^. Implementing the developed prototype for industrial wireless sensor and actuator networks (IWSAN) in Yu et al.’s study showcases the potential of IWSANs to provide stable and real-time transmission for many industrial applications in different working environments^27^. The prototype’s centralized management easily handles network activities, such as scheduling, routing, and time synchronization management. The study also shows that the average packet delivery ratio with the deadline constraint is significantly high, achieving a packet delivery measurement for uplink and downlink of 97.5%, higher than TDMA and flooding mechanism. However, the performance is not compared with wired NCS solutions, which will provide a strong basis for evaluating the effectiveness of the wireless solution.

Empirical studies reveal that graph routing offers better worst-case reliability than source routing, albeit at the expense of increased latency, effective process monitoring, control, and energy consumption^28^. The study reveals that channel hopping mitigates the erratic nature of transmission failures. In contrast, a considerable distance between channels can lessen the consecutive transmission failures amongst links that share a standard receiver. Empirical evidence confirms the efficacy of the proposed algorithm in enhancing the network’s overall performance. However, the study did not account for the impact of packet loss, real-time process control, interference, network size, and topology on the algorithm’s performance. Researchers in^29^ proposed the Internal Model Control approach as an alternative method for compensating delay variations in wireless processes compared to the traditional PID control structure. The study involves simulations conducted on industrial benchmark process models of first, second, and third-order. Results obtained from a real-time WirelessHART hardware-in-the-loop simulator reveal that the proposed approach demonstrates greater robustness to delay network variation than the PID. It is essential to note that the simulations were conducted without considering any process dead-time conditions, which raises concern about the effectiveness of the delay compensation in the controller.

In a study by Liu et al., a process control system with 41 sensors and 12 actuators is used to manage production at the Tennessee Eastman Challenge Model plant^30^. The system is decentralized and compared the performance of wireless and wired links. The wired links provided instant and 100% reliable communication. Nevertheless, the study did not account for the potential impact of other wireless communication factors, such as process dead-time, jitter, and security, which may also influence the overall control performance. The study conducted by Anders et al.^31^ demonstrated that wireless control systems can effectively replace wired ones, even in complex industrial settings. Researchers conducted a study on a paper mill’s starch cooker processes using wireless technology. The authors have used standard PID controllers and included wireless sensors and actuators with a failure detection feature. The tests showed successful results, proving the potential of wireless technology in next-generation process control.

Hasan et al. proposed the utilization of PI-PD and FOPI-FOPD controllers in wireless NCS to address packet loss and enhance system performance effectively^32^. In high packet loss scenarios, a PI controller is introduced as a compensator in the feed-forward loop to retain system stability. The wireless NCS is simulated using MATLAB/Simulink and TrueTime simulator, and the grey wolf optimization algorithm determines the optimal controllers and compensator parameters. In Ref.^33^, the design of controllers for linear time-invariant systems that experience correlated random packet losses during communication with actuators. These packet losses are modelled as a finite-length Markov chain. The authors propose a method that utilizes the problem’s structure to design an optimal controller. However, storing optimal control laws requires exponentially more space when considering longer packet loss histories. Han et al. researched the optimal output feedback control for NCS that experience Markovian packet losses^34^. A two-state Markov chain is used to describe the packet loss channels, and the study adopted the dynamic programming approach to derive the optimal output feedback control based on the solution to a modified Riccati equation. This approach is an essential implementation of control theory for NCS with unreliable communication channels. The optimal control strategy derived from the modified Riccati equation is based on the separation principle, resulting in an optimal recursive estimator. A scalable method for system analysis and controller synthesis for homogeneous multi-agent systems with Bernoulli distributed packet loss is proposed in Ref.^35^. The approach, formulated in terms of linear matrix inequalities independent of the number of agents, offers wide applicability. A numerical first-order consensus example is used to demonstrate the effectiveness of the proposed techniques. The upper bounds on the H2-norm obtained through the method are compared to estimates from the Monte-Carlo simulation.

There have been significant improvements to the conventional PI controllers, resulting in the development of several types of controllers, including fuzzy PI FOPI, SWPI, PPI, and NPI^36–40^. These controllers are particularly useful for managing processes with longer dead-time. However, advanced control techniques have also been used to enhance the performance of PI controllers. These approaches, including dead-time compensators, model predictive, internal model, and generalized predictive controllers, often have complex designs and require more tuning parameters, making them unsuitable for industrial processes^29,41^. Furthermore, many controllers require an exact process model to optimize control^16^. Managing dead-time processes in industrial environments is challenging due to the inherent time delay that characterizes these systems^42,43^. It is essential to note that conventional PI controllers will perform poorly in the closed-loop control system, which can significantly affect the system’s overall performance^26^. This can be attributed to increased phase lag resulting from time delays. Iterative learning control is an effective approach proposed for process plants with dead-time, with the primary objective of improving the closed-loop performance in the presence of high disturbances and set-point variations^44^. The designed controller’s robustness and efficiency in producing faster convergence are achieved through continuous output tracking, leveraging the reference plant model. The ILC design process begins with designing L- and Q-filters for the controller using the process model. After error reduction and control signal conditioning, the obtained filter coefficients are then integrated and fed to the feed-forward path. This integration leads to an overall better performance with a reliable solution to improve the performance of process plants with dead-time. Its effectiveness in mitigating the adverse effects of disturbances and set-point variations makes it a valuable tool for enhancing the closed-loop performance of such systems.

Researchers have developed a new predictive PI controller to address these issues that combines conventional PI and Smith predictor^45^. Although this controller may not achieve robust performance during significant disturbances and may show poor performance due to the divergence between the real and designed models, it represents a significant step forward^46^. However, inconsistencies between the process plant’s real-time performance and the predictive PI controller will arise due to model mismatch. Such differences cause inadequate closed-loop performance, which necessitates the deployment of a reliable controller to enhance the performance of dead-time process plants^47^. Helber et al.^48^ proposed a system design for high-order and fractional-order processes. This design employs controllers of the FOPID and FOPI and considers the delicate balance between robustness and performance. The simulations’ results indicate that the proposed tuning approach enhanced the load disturbance rejection, set-point tracking and controllability. In all the above literature, the performance of the FOPI controller is suboptimal in processes with extended dead-times, exhibiting sluggish and oscillatory responses. As such, there is considerable scope for enhancing both conventional and advanced controller performance in such conditions. Hence, this paper proposes a robust fractional-order predictive PI (FOPPI) controller, which only needs the system model of the considered process and addresses these challenges by integrating the conventional FOPI controller with the dead-time compensating Smith predictor. The resulting hybrid controller offers a superior solution to the limitations of traditional FOPI controllers in longer dead-time processes.

**Figure 1:**
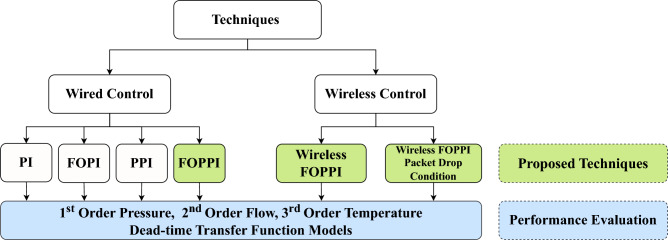
Overall research flow of the article.

The overall flow of this article is shown in Fig. 1. The research article presents several notable contributions, which can be summarized as follows:

1. The proposed fractional-order predictive PI (FOPPI) compensator is the foremost solution for wired and wireless networks facing prolonged dead-time processes.
2. At first, the controllers are set up on wired networks. Then, the most effective FOPPI controller is used for wireless network control.
3. According to performance metrics such as disturbance rejection and set-point tracking, it has been observed that the FOPPI controller surpasses conventional controllers.
4. The FOPPI controller has been tested in various benchmark process models and has proven to reduce peak overshoot, which maximizes the operating lifespan of control valve actuators.
5. In wireless networks, the FOPPI controller has exhibited superior set-point tracking and faster rise time performance, highlighting its robust control capabilities. Also, the controller is tested for various packet drop scenarios from 70 to 90%.
6. Even in a packet loss scenario, the FOPPI controller has shown its ability to control the process without access to the complete process variable. The results indicate that the FOPPI can maintain process stability with only 30% of the available process data.

## Methodology

This section initially provides an understanding of both traditional PI and fractional-order PI controllers, along with the equations that define them. Secondly, the proposed dead-time compensating fractional-order predictive PI controller’s detailed design and implementation is given. The designing is carried out using the Smith predictor, and the FOPDT technique is discussed. Lastly, the derivation process of the new controllers from conventional PI is explained with their pictorial representation.

### Fractional-order PI

**Figure 2:**
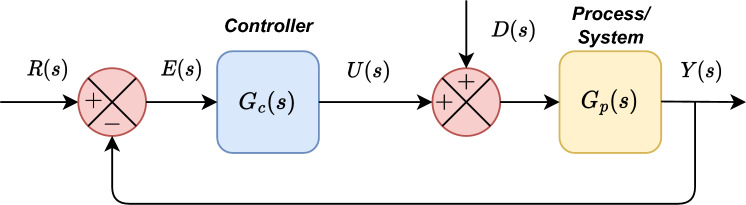
Block diagram of a closed-loop feedback control system.

In closed-loop systems, the unity feedback configuration, illustrated in Fig. 2, comprises several important process variables. These variables include the controller ($G_c(s)$), the process plant ($G_p(s)$), the set-point (*R*(*s*)), the output response (*Y*(*s*)), the error (*E*(*s*)), the controller signal (*U*(*s*)), and the external disturbance is (*D*(*s*)). Each of these variables plays a critical role in the overall functionality and efficacy of the closed-loop system. Understanding the significance and interplay of these variables is key to optimizing performance and ensuring successful outcomes.

Let $G_c(s)$ be the PI controller in the given block diagram. Then the control signal of the PI controller is expressed as follows:

1 $$\begin{aligned} U(s)= K_p\Bigg (1+\frac{1}{T_is}\Bigg )E(s) \end{aligned}$$

The controller’s proportional gain and integral time constant are denoted by $K_p$ and $T_i$, respectively. The integral action of the conventional PI controller is fractionalized using $\lambda$ to obtain a fractional-order PI controller. The above mentioned Eq. (1) is used to obtain the resulting fractional-order PI (FOPI)’s signal as,

2 $$\begin{aligned} U(s)=K_p\bigg (1+\frac{1}{T_is^\lambda }\bigg )E(s),~0<\lambda <1 \end{aligned}$$

Error signal *E*(*s*) shown in Eqs. (1) and (2) is obtained as follows,

3 $$\begin{aligned} E(s) = R(s) - Y(s) \end{aligned}$$

### Fractional-order predictive PI

In order to accurately determine the control signal for the fractional-order predictive PI (FOPPI) controller, it is imperative to take into account the transfer function of $G_p(s)$ as a First Order Plus Dead-Time (FOPDT) process within the block diagram. This crucial step is to include the essential process dynamics for developing an effective control strategy with better dead-time compensation ability. The FOPDT equation is given as,

4 $$\begin{aligned} G_p(s)=\frac{K}{1+Ts}e^{-sL_p} \end{aligned}$$

The equation mentioned above defines the essential variables of the FOPDT system, including the process gain, dead-time, and time constant, denoted as *K*, $L_p$, and *T*, respectively. However, it is essential to acknowledge that determining these parameters can be complex and time-consuming, as Fabio Peterle et al. in their study on processes with dead time^49^. An analysis of the block diagram reveals a clear relationship between the closed-loop transfer function $G_o(s)$ of *R*(*s*) and *Y*(*s*) as,

5 $$\begin{aligned} G_o(s)=\frac{Y(s)}{R(s)}=\frac{G_c(s)G_p(s)}{1+G_c(s)G_p(s)} \end{aligned}$$

In order to derive the controller $G_c(s)$, it is necessary to rearrange the above equation in the following way,

6 $$\begin{aligned} \frac{G_p(s)}{G_o(s)}= & {} \frac{1+G_c(s)G_p(s)}{G_c(s)} =\frac{1}{G_c(s)}+\frac{G_p(s)G_c(s)}{G_c(s)}=\frac{1}{G_c(s)}+G_p(s) \end{aligned}$$

7 $$\begin{aligned} \frac{1}{G_c(s)}= & {} \frac{G_p(s)}{G_o(s)}-G_p(s) =\frac{G_p(s)-G_p(s)G_o(s)}{G_o(s)} \end{aligned}$$

8 $$\begin{aligned} G_c(s)= & {} \frac{U(s)}{E(s)}=\frac{G_o(s)}{G_p(s)\big (1-G_o(s)\big )} \end{aligned}$$

In the forthcoming mathematical equation, it is pertinent to note that the closed-loop transfer function has been designated as $G_o(s)$ to facilitate understanding.

9 $$\begin{aligned} G_o(s)=\frac{1}{1+Ts}e^{-sL_p} \end{aligned}$$

Obtaining the controller transfer function is a straightforward process by substituting Eqs. (4) and (9) into Eq. (8). Further steps have been provided below to achieve $G_c(s)$ as,

10 $$\begin{aligned} G_c(s)= & {} \frac{1}{\frac{K}{1+Ts}e^{-sL_p}}\bigg (\frac{\frac{1}{1+Ts}e^{-sL_p}}{1-\frac{1}{1+Ts}e^{-sL_p}}\bigg ) \end{aligned}$$

11 $$\begin{aligned} G_c(s)= & {} \frac{U(s)}{E(s)}=\frac{1+Ts}{K(1+Ts-e^{-sL_p})} \end{aligned}$$

The FOPPI’s control signal *U*(*s*) is achieved by expressing the above $G_c(s)$ using input-output relation as,

12 $$\begin{aligned} U(s)=K_p\bigg (1+\frac{1}{T_is^\lambda }\bigg )E(s)-\frac{1}{T_is^\lambda }(1-e^{-sL_p})U(s),~0<\lambda <1 \end{aligned}$$

Using the FOPPI controller, as given in Eq. (12), necessitates the relationship of the proportional gain $K_p$ as the inverse of the process gain *K*, i.e. $K_p=\frac{1}{K}$. Moreover, the integral time $T_i$ is the equivalent value of the process time constant *T* to meet the desired process outcomes, while $\lambda$ is the integration order.

**Figure 3:**
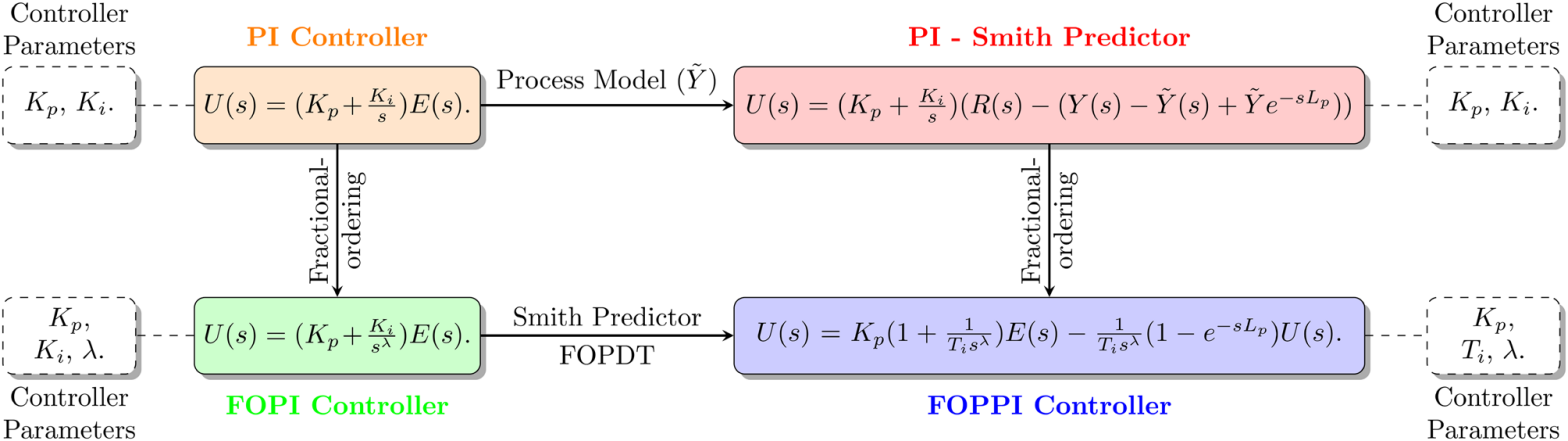
Evolution of the fractional-order controllers.

The diagram depicted in Fig. 3 portrays the progressive development of conventional PI controllers into FOPI, Smith predictor PI, and FOPPI controllers. In this evolution, the empirical model of the FOPDT is much more essential in solving the issues that occurred in the dead-time processes by compensating them in the feedback loop. This is clearly observed in the Smith predictor, and the figure shows the FOPPI control signal *U*(*s*). Additionally, the figure showcases the integrated construction of the proposed controller, which involves combining the essential process variables from both FOPI and Smith predictor PI controllers. Further, the number of controller parameters is also given in every controller.

**Figure 4:**
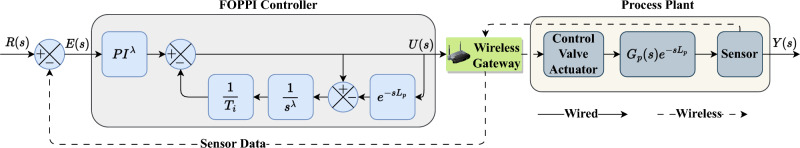
Fractional-order dead-time compensator in the wireless network.

Figure 4 shows a visual illustration of the effectual implementation of the proposed FOPPI controller. Here, the FOPPI is implemented on the wireless closed-loop sensor network. In this process, the controller will receive and send the process and control signal from the sensor and actuator wirelessly using the gateway having IEEE 802.15.14 communication protocol. The process signal from the gateway is directly fed to the controller along with the input signal to the control loop for noise removal and signal conditioning.

## Results and discussion

This section will begin with an introduction to the selected process models, which mimic industrial-scale process control operations. Secondly, various metrics to examine the controller’s performance is discussed. Thirdly, a comprehensive analysis is conducted, which incorporates a comparison of the FOPPI controller with other commonly used controllers such as the PI, FOPI, and PPI controllers. This analysis will also take into consideration both wired and wireless network environments. The simulations were conducted using MATLAB/Simulink software (2021a) on a 3.10 GHz Intel(R) Xeon PC with 16.00 GB of RAM. The setup allowed for efficient and accurate execution and all the parameters used for the simulations are given in the Tables 1, 2, and 3.

### Process model

This study utilizes highly accurate real-time non-linear dead-time process models to simulate various industrial plants’ dynamic behaviour for different controllers. These models are derived from actual industrial processes, including a thermal chamber model, a third-order process utilized by Tan et al.^50^, and a first-order pressure regulation process and second-order flow control process employed by Arun et al.^42^. Utilizing the following real-time processes transfer function models in simulations is a highly effective tool for gaining insights into the intricate behaviour of industrial plants.

13 $$\begin{aligned} G_p(s)= & {} \frac{0.866e^{-s}}{1.365s+1} \end{aligned}$$

14 $$\begin{aligned} G_2(s)= & {} \frac{1.3e^{-5s}}{s^2+2s+1} \end{aligned}$$

15 $$\begin{aligned} G_3(s)= & {} \frac{e^{-5s}}{s^3+3s^2+3s+1} \end{aligned}$$

**Figure 5:**
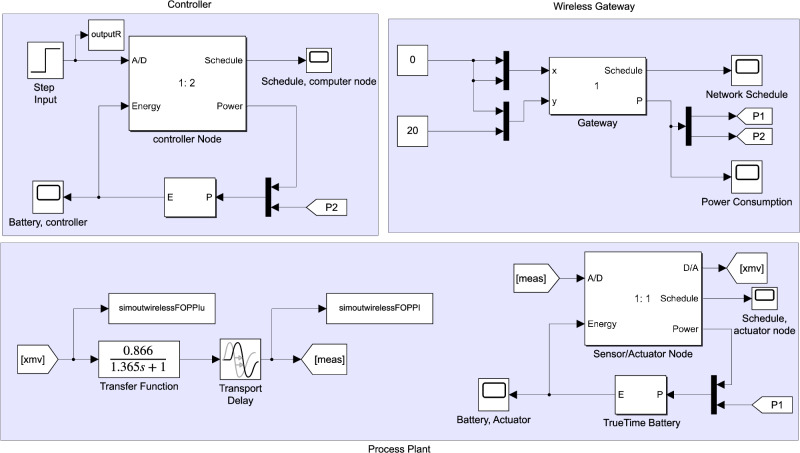
Simulation of the process plants using the proposed FOPPI on the wireless network in MATLAB/Simulink.

**Figure 6:**
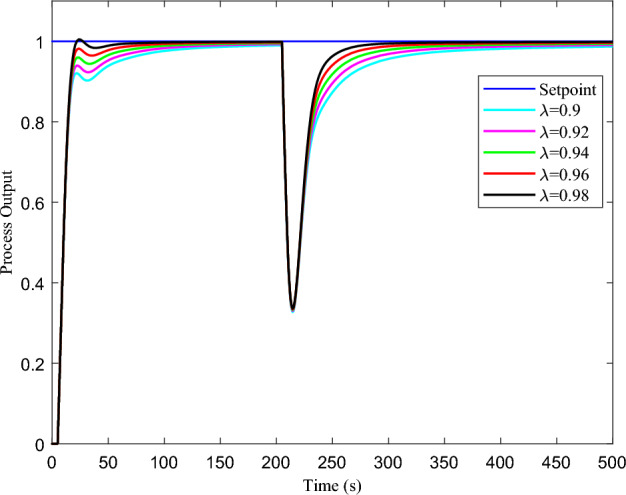
Performance of the FOPPI controller for different values of $\lambda$.

The proposed study uses conventional techniques to derive controller parameters for easy and better turning. Specifically, transfer function parameters of the first-order systems given in Eq. (13) are used to obtain the controller parameters directly. At the same time, the auto-tuning feature from the Simulink toolbox will be implemented for second and third-order systems. This research seeks to optimize the control of systems and provide a more efficient and practical approach to control systems while adhering to the highest standards of the process settings. The resulting parameters are presented in Tables 1, 2 and 3. Implementing the proposed FOPPI controller in wireless networks is carried out utilizing MATLAB/Simulink using the TrueTime toolbox, as depicted in Fig. 5. The implementable FOPPI controller structure heavily relies on the use of the fractional-order integrator, denoted as $1/s^\lambda$ in Eq. (12). The Oustaloup approximation technique is one of the most widely used approximation methods to obtain the fractional-order^51, 52^. Thus, the FOPPI integrator with optimal parameters ($\omega _b$,$\omega _h$) = ($10^{-5},10^{5}$) and $N=5$ is used to obtain the fractional parameter ($\frac{1}{s^{0.98}}$). This approximation results in a transfer function for the fractional-order integrator is given in Eq. (16). Further, the choice of selecting the fractional-order integer ($\lambda$)=0.98 for the process models based on the trial and error method is given in Fig. 6.

16 $$\begin{aligned} \frac{1}{s^{0.98}}\approx \frac{\big [ 871s^5 + 6.03\times 10^4 s^4 + 2.478\times 10^5 s^3 + 6.398\times 10^4 s^2 + 1038\times s + 1\big ] } {\big [s^5 + 1038 s^4 + 6.398\times 10^4 s^3 + 2.478\times 10^5 s^2 + 6.03\times 10^4 s + 1\big ]} \end{aligned}$$

### Performance metrics

An in-depth analysis of the simulation results of the above real-time process models regarding their performance under specific conditions has been conducted. Factors such as rise time, settling time, overshoot, set-point tracking, disturbance rejection, and noise reduction have been analyzed and used for the comparison. Also, the controllers are compared with different error performance criteria such as integral square error (ISE), integral absolute error (IAE), and integral time absolute error (ITAE) to understand the effectiveness of minimizing the process error value. In these error performance criteria, the controller with lease value signifies the ability to minimize the error value, leading to better performances. A disturbance of 50% is injected into the control signal for all the processes at various times. After comparing the wired PI, FOPI, PPI, and PPI controllers, the FOPPI controller outperformed the others, so it is implemented as the controlling element in the wireless control. Furthermore, it has been preset that 70% packets will be deliberately dropped during transmission to the controller for the packet drop analysis. The primary goal of this investigation is to assess the FOPPI controller’s ability to effectively stabilize and compensate for the dead-time process, even when only 30% of the process data is available.

### First-order system

**Figure 7:**
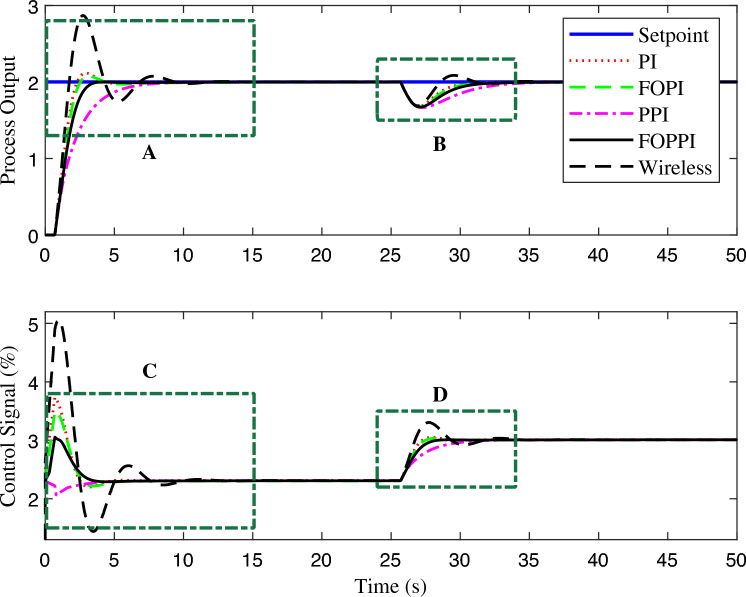
First-order system disturbance rejection analysis of different controllers.

**Figure 8:**
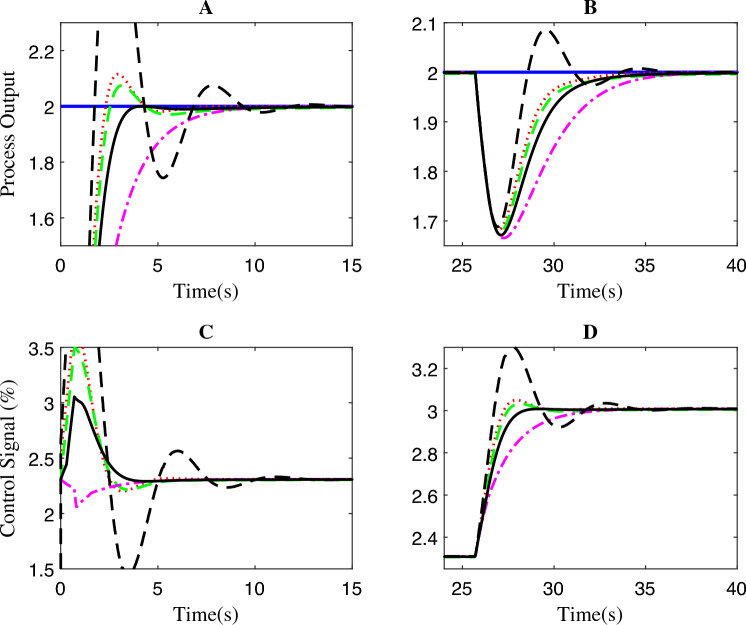
Zoomed region of Fig. 7.

**Figure 9:**
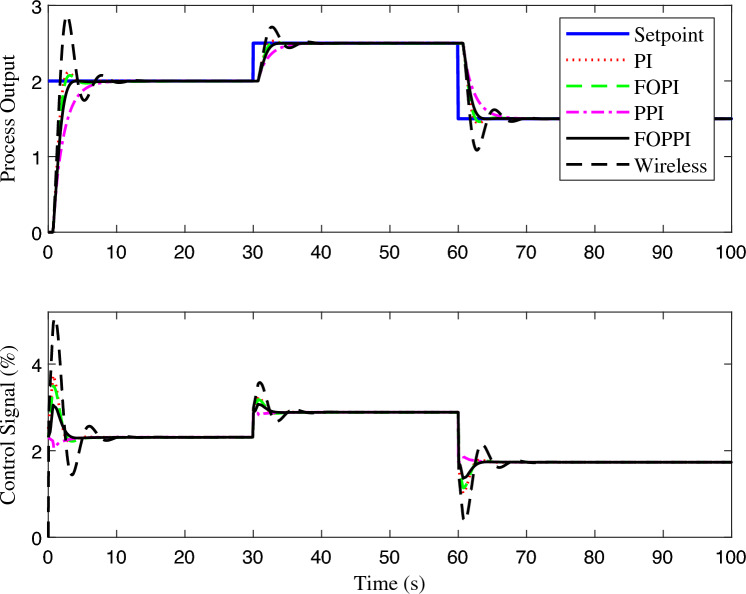
First-order system set-point tracking performance of the various comparing controllers.

**Figure 10:**
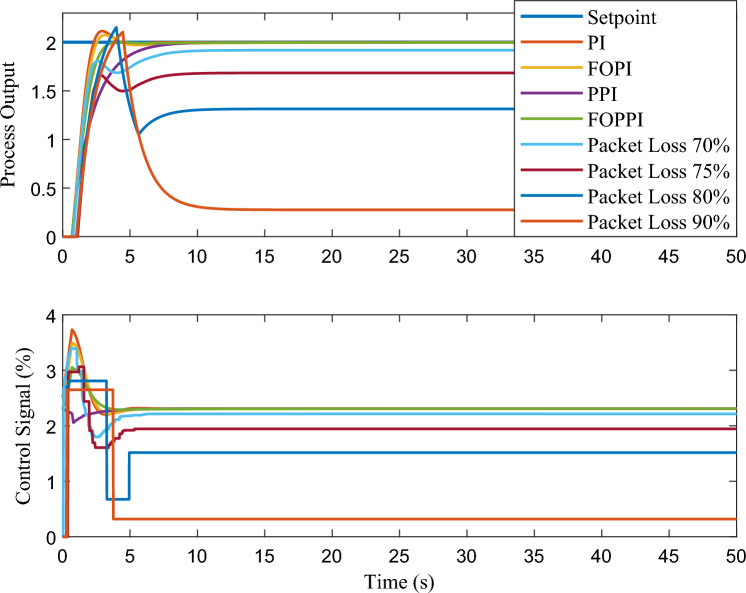
Performance of wireless control on first-order system for the packet drop scenario.

This subsection presents the simulation results of the pressure process system, as expressed in Eq. (13). The corresponding performance comparisons are depicted in Figs. 7, 8, 9, and 10. The specified regions of interest, denoted as A, B, C, and D, have been utilised for the purpose of performing a detailed analysis of the zoomed regions. The performance evaluation has been quantitatively assessed, and the results are presented in Table 1.

**Table 1:** Performance of various controllers and its parameters in the first-order system.

**Controller**	$K_p$	$K_i$	$\lambda$	$t_r$	$t_{s1}$	$t_{s2}$	%OS	ISE	IAE	ITAE
PI	1.153	0.846	–	1.1636	7.4218	30.8282	23.4846	5.255	4.065	155.2
FOPI	1.153	0.846	0.98	1.2832	7.5106	30.7285	22.4738	5.645	4.273	168.4
PPI	1.153	0.846	–	3.4885	8.9305	34.7421	0.00015	6.075	5.836	250.3
FOPPI	1.153	0.846	0.98	1.6739	5.4801	30.6817	0.0750	4.74	3.711	129.7
Wireless	1.153	0.846	0.98	0.8115	10.6886	35.4969	43.4261	81.7	35.6	564.95
Packet loss (70%)	1.153	0.846	0.98	0.8020	55.6067		0.00012	273.6	154.5	1256.645

The controllers’ disturbance rejection performance is presented in Fig. 7. Evidently, the wireless FOPPI controller outperformed all other controllers with a remarkable rise time of 0.702 s, which is 2.7865 s faster than the slowest PPI controller, resulting in a significant 329.883% performance increment. It is worth noticing that the wired PI, FOPI, and dead-time compensating FOPPI controllers had comparatively slower rise times of 1.1636, 1.2832, and 1.6739 s, respectively. At the 25 s, a disturbance of 50% is introduced, and the subsequent performance demonstrated that most controllers are able to restore and stabilize at the intended set-point value successfully. The proposed FOPPI controller outperformed the slowest wireless control in both settling time performances.

Significantly, the FOPPI settled with the values of 5.2085 s and 4.8152 s faster, respectively, for before and after disturbance $$(t_{s1}$$ and $$t_{s2})$$, which is notably quicker than the wireless. Although the rise time is faster in wireless, as shown in Fig. 8, region A, it is noteworthy that this scenario did not lead to improved performance and resulted in a substantial sluggish settling time of 10.6886 s and 35.4969 s, respectively. The conventional dead-time compensator PPI faced significant issues with the sluggish rise time of 3.4885 s. As a result, the settling time is almost similar to the wireless control, which is 8.9305 s at $$t_{s1}$$ and 34.7421 s at $$t_{s2}$$. During the settling time after the disturbance, it can be noted that PI, FOPI, and FOPPI displayed almost identical setting values. However, the FOPPI displayed a slightly faster settling compared to the others. It is evident from the results illustrated in Fig. 8 for region B that the PPI faced more challenges while handling the external disturbance, resulting in a comparatively delayed settling time in this particular scenario. Considering the PPI controller, it had the smallest %OS of 0.00015%. However, the wireless FOPPI had a significantly larger overshoot value of 43.4261%, which can directly affect the control valve actuator’s lifespan, throughput and efficiency. At the same time, the wired FOPPI had the second lowest %OS value of 0.0750% among all the controllers, followed by FOPI at 2.4738% and PI at 23.4846%. These results demonstrate the superior performance of the proposed FOPPI controller. It should be noted that the wireless FOPPI’s control signal in regions C and D of Fig. 8 exhibited the fastest response starting at 0.3 itself, leading to quicker rise time performance. In the error performance criteria, the proposed controller has the least error values of 4.74, 3.711, and 129.7 in ISE, IAE, and ITAE, respectively.

The proposed FOPPI achieved remarkable ability to track set-point changes, as evident from the variable set-point tracking performance shown in Fig. 9. Conversely, the wireless FOPPI had a significant weakness in tracking the set-point, which resulted in an initial higher overshoot and sustained deviation offset of the process and control signal. Here, the PPI’s control signal showed a declining trend, which led to the sluggish rise and settling time performances. The remaining PI and FOPI controllers followed the set-point at different speed paces, while FOPPI had a lower initial shoot-up showing its robustness. In the packet loss condition of the wireless FOPPI controller, it managed to track the set-point with the initial sensor/actuator data, leading to a quicker rise time. However, after the packet loss of 70% during every second, causing its control signal to deteriorate and take the control actions with its only known process value data of 30%. This sluggishness can be noticed in the control action in Fig. 10. After the absence of a constant amount of process data, the controller tends to saturate less than the desired set-point with a higher offset than the allowed range in the process control resulting in undesired output. However, this scenario did not lead to process instability, showing the FOPPI controller’s robustness in maintaining process integrity in wireless networks.As packet loss reaches the 70% threshold, the system’s response becomes noticeably slower and more inconsistent. At 80% packet loss, the controller attempted to take control actions with a faster rise time than the PPI, but these actions were short-lived and resulted in drastic process response outputs. By the time the packet loss reached 90%, the system had become uncontrollable, with the controller only achieving a process variable value of 0.5, which highlights the significant impact of packet loss on the system’s controllability.

### Second-order system

**Figure 11:**
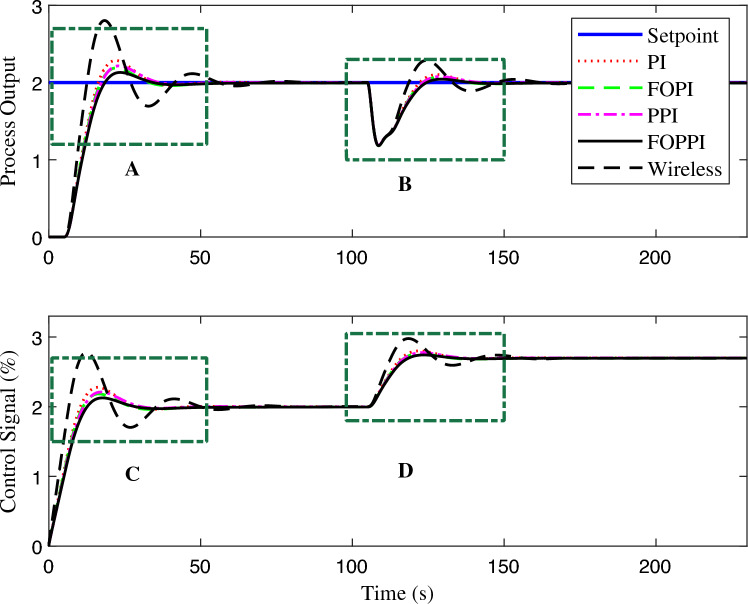
Second-order system disturbance rejection analysis of different controllers.

**Figure 12:**
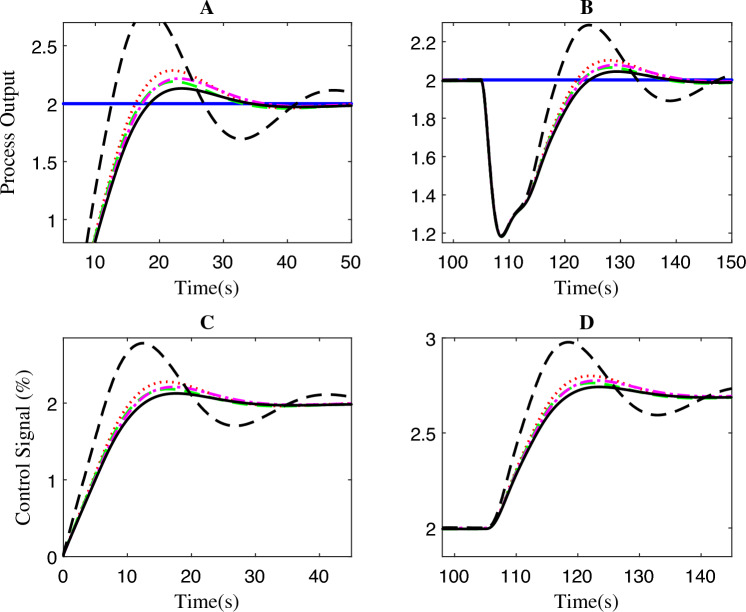
Zoomed region of Fig. 11.

**Figure 13:**
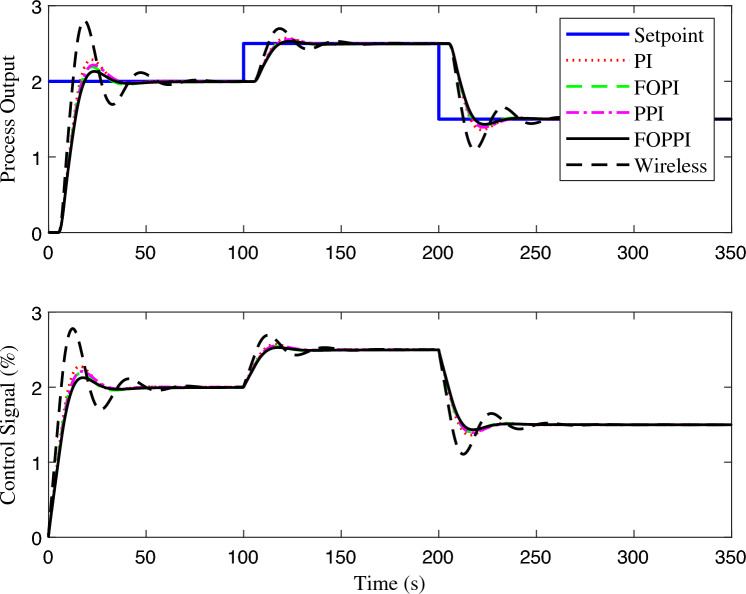
Second-order system set-point tracking performance of the various controllers.

**Figure 14:**
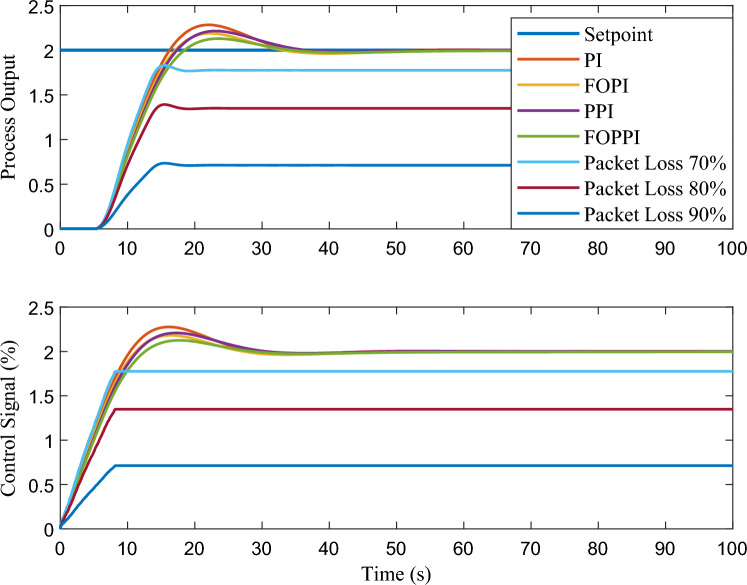
Performance of wireless control on second-order system for the packet drop scenario.

This subsection presents the system simulation results expressed in Eq. (14). The performance comparisons are shown in Figs. 11, 12, 13, and 14. Like the first-order process, this system also compares performance with multiple zoomed regions of interest for better analysis. The respective controller parameters and corresponding numerical analysis results are tabulated in Table 2.

**Table 2:** Performance of various controllers and its parameters in the second-order system.

Controller	$K_p$	$K_i$	$\lambda$	$t_r$	$t_{s1}$	$t_{s2}$	%OS	ISE	IAE	ITAE
PI	0.0124	0.106	–	7.6112	33.9700	135.3501	14.3002	47.431	36.581	5171.66
FOPI	0.0124	0.106	0.98	8.0627	32.7790	133.3292	9.7129	48.065	37.989	5196.21
PPI	0.0124	0.106	–	8.2931	34.2367	135.4559	10.8101	50.641	47.198	5434.47
FOPPI	0.0124	0.106	0.98	8.3003	30.1915	130.1851	6.7409	36.813	35.984	5006.92
Wireless	0.0124	0.106	0.98	5.0014	60.6617	153.1894	37.5150	190.65	708.79	10788.19
Packet loss (70%)	0.0124	0.106	0.98	6.1302	157.5020		0.00013	387.46	1796.8	22309.85

The evaluation of the system’s performance shown in Fig. 11 yielded noteworthy results indicating that the wireless FOPPI demonstrated the fastest rise time, achieving its target in a mere 5.0014 s, showing a 65.9595% quicker response time to the initial set-point changes. Conversely, the slowest FOPPI took a considerably longer time of 8.3003 s. Moreover, the conventional PI exhibited a response time of 7.6112 s, while the FOPI and PPI had rise times of 8.0627 s and 8.2931 s, respectively. The FOPPI controller demonstrated exceptional settling time performance, outpacing the wireless FOPPI by settling almost two times faster and achieving a 100.923% increase in performance as seen in Fig. 12 regions A and B. Unfortunately, the wireless FOPPI suffered a setback in this performance, settling almost twice slower than the other controllers at 60.6617 s.

The conventional dead-time compensator PPI had the second slowest settling time at 34.2367 s, followed by PI at 33.9700 s. It is worth noting that the FOPI performed almost as impressive as the proposed FOPPI, settling only 2.5875 s apart. In the settling performance after the disturbance rejection, the wireless FOPPI managed to settle at 153.1894 s. Despite the slowest settling time, it avoided the significant time difference experienced in the previous case. Here also, the FOPPI secured first place by settling 5.2744 s ahead of the existing PPI controller, which settled at 135.4559 s. The traditional PI and FOPI controllers settled at 135.3501 and 135.4559 s, respectively. The proposed FOPPI demonstrated a minimal overshoot performance with a value of 6.7409%.

However, FOPPI in wireless networks, the minor process signal delay during transmission accumulates over time, resulting in a significant increase in peak overshoot of 37.5150% which resulted in 82.0315% decreased performance compared to wired FOPPI control highlighting the impact of minor delays in wireless networks. Furthermore, the FOPI had the second-lowest value of 9.7129%, while PPI and PI had respective values of 10.8101% and 14.3002%. Upon analyzing the control actions in regions C and D of Fig. 12, it is evident that all controllers were initialized at the origin. However, it is observed that the wireless FOPPI exhibited superior set-point tracking ability, leading to a faster rise time. On the other hand, the wired FOPPI demonstrated the most linear trend that followed the process variable’s pattern. This is true in the case of PI, FOPI, and PPI, where they had a minor overshoot in their control signals. The set-point tracking response of the second-order system is shown in Fig. 13. Due to the system’s longer dead-time, the controllers’ time to reach and track the set-point are synchronized with each other. It is important to note that the wireless FOPPI’s overshoot is minimized during consecutive set-point changes, demonstrating its ability to handle load variations resulting from set-point variation. At the same time, the other controllers displayed varying speeds in achieving the desired set-point, with most of them showing relatively closer results.

The effectiveness of closed-loop control over the packet loss condition of the wireless FOPPI controller is illustrated in Fig. 14. All the controllers successfully tracked the set-point with the initial sensor/actuator data, resulting in a quicker rise time. However, it is worth noting that the control actions of the FOPPI controller became saturated at 1.7%, leading to more offset settling with the process variable. Nevertheless, like the first-order system, the wireless FOPPI’s control actions maintained the stability of the process without causing unsafe conditions. As the system reaches a value beyond 70%, its controllability gradually diminishes due to the unavailability of process data to the FOPPI controller. Additionally, the control actions of the FOPPI get saturated after a certain period, leading to increased offset and undesired process response.

### Third-order system

**Figure 15:**
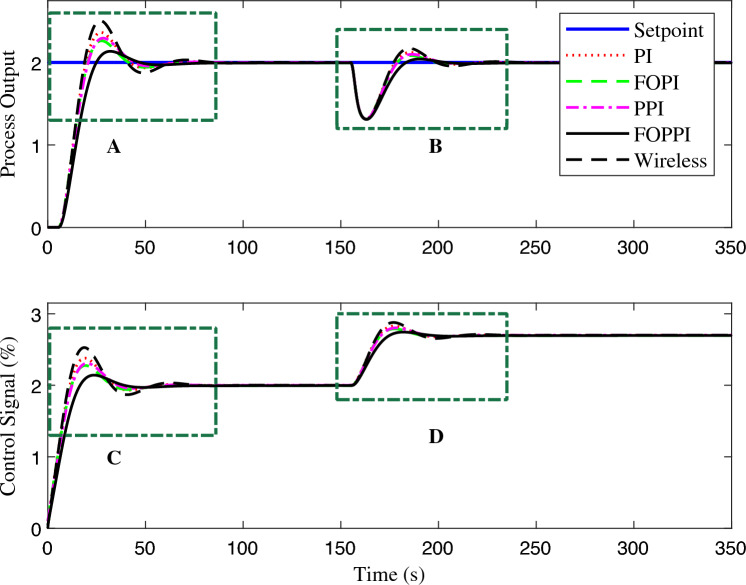
Third-order system disturbance rejection analysis of different controllers.

**Figure 16:**
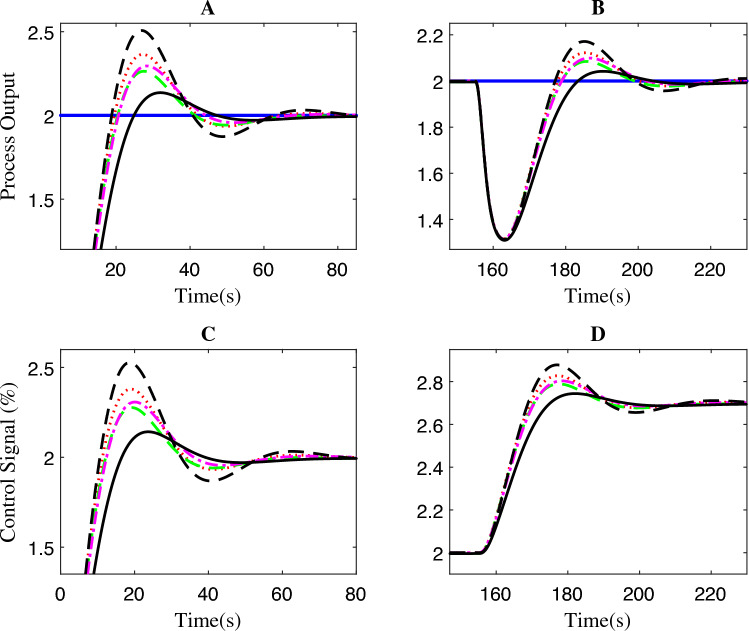
Zoomed region of Fig. 15.

**Figure 17:**
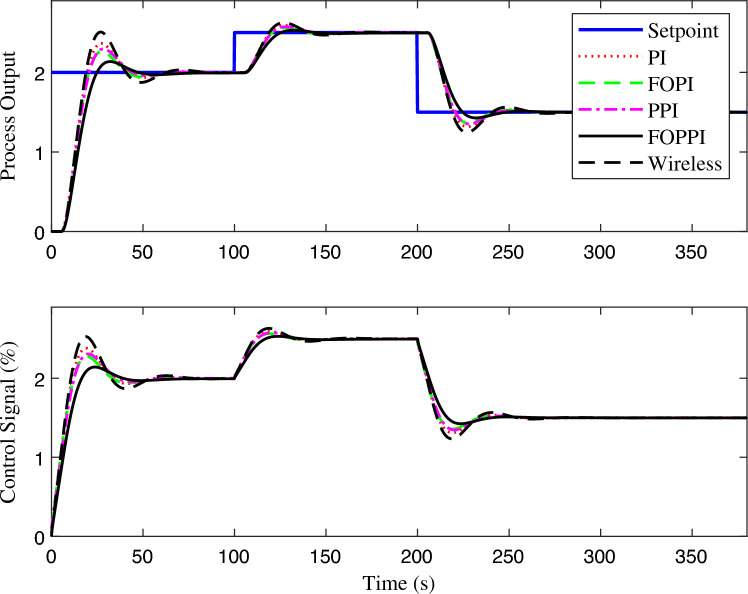
Third-order system set-point tracking performance of the various controllers.

**Figure 18:**
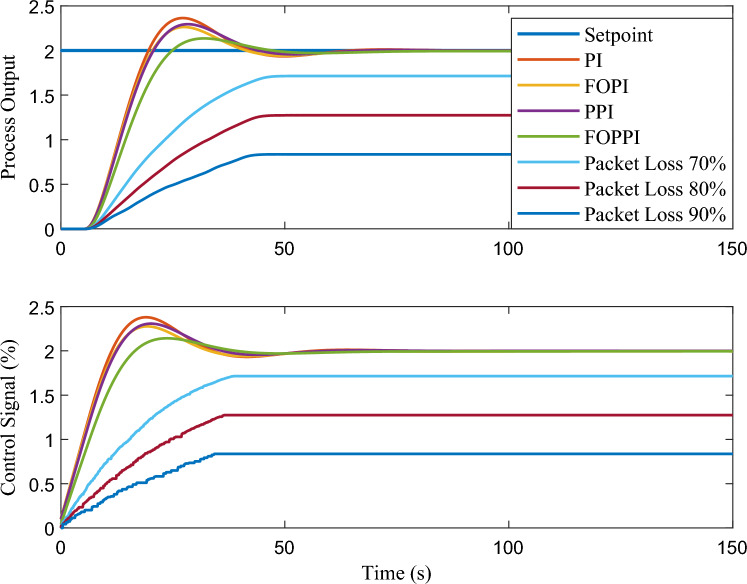
Performance of wireless control on third-order system for the packet drop scenario.

Similarly to the first and second-order systems, the third-order system thoroughly examines the simulation outcomes of the process transfer function given in Eq. (15). The quantitative assessment of the performance evaluation is presented in Table 3. The performance comparisons of these scenarios are illustrated in Figs. 15, 16, 17, and 18. The specified areas of interest, marked as A, B, C, and D, have been used to analyse the zoomed regions comprehensively.

**Table 3:** Performance of various controllers and its parameters in the third-order system.

Controller	$K_p$	$K_i$	$\lambda$	$t_r$	$t_{s1}$	$t_{s2}$	%OS	ISE	IAE	ITAE
PI	0.0202	0.0819	–	9.7231	66.4610	217.5030	18.209	53.95	43.25	16727.88
FOPI	0.0202	0.0819	0.98	10.259	65.8812	216.8085	13.338	50.45	42.97	14545.95
PPI	0.0202	0.0819	–	10.462	64.3925	215.3418	14.767	49.58	42.16	13765.03
FOPPI	0.0202	0.0819	0.98	11.814	52.8923	201.5863	6.968	43.06	38.15	12006.98
Wireless	0.0202	0.0819	0.98	9.0034	68.7303	219.4727	24.355	394.08	197.11	365082.2
Packet loss (70%)	0.0202	0.0819	0.98	27.928	254.3092		0.0	483.37	364.98	78654.06

The wireless FOPPI outperformed its wired counterparts regarding disturbance rejection, as shown in Fig. 15. With a rise time of 9.0034 s, it achieved a remarkable improvement of 2.8106 s over the slowest wired FOPPI. It is worth noting that the traditional PI controller showed a very similar result to the wireless control, with only a slight difference of 0.7197 s. Moreover, both FOPI and PPI controllers performed similarly in terms of rise time, reaching them in 10.259 s and 10.462 s, respectively. Lastly, the wireless FOPPI controller with packet loss condition had a sluggish rise of 27.928 s, securing the last position. While analyzing the zoomed Fig. 16 region B, it is evident that the wired FOPPI controller exhibits the most optimal and linear recovery from the disturbance with minimal offset, surpassing the other controllers. This is supported by the numerical analysis in $t_{s2}$, which shows that the proposed controller settles 17.8864 s ahead of the wireless FOPPI controller, which settled at 219.4727 s.

The PPI controller follows in second place, settling at 215.3418 s, but is 13.7555 s slower than the FOPPI. The FOPI and PI controllers follow in succession, with settling times of 216.8085 s and 217.5030 s, respectively. Notably, the wireless FOPPI control settles almost as closely with the existing PI controller at 219.4727 s in $t_{s2}$, with a difference of only 1.9697 s, demonstrating the effectiveness of using the controller for higher-order systems. In the peak overshoot performance, the wired FOPPI once again produced the most negligible overshoot value of 6.968%, resulting due to its robust and improved control signal, which can be seen in regions C and D of Fig. 16. In the meantime, the FOPI yielded second position with the value of 13.338%, which is a 91.4179% increase in the overshoot while comparing the FOPPI. The PPI and PI follow the third and fourth positions, where the wireless FOPPI produced the highest overshoot of 24.355%. Despite the highest value in this process, the wireless FOPPI produced the least overshoot values compared to its previous first and second-order results. The set-point tracking response of the third-order system is illustrated in Fig. 17. This performance follows a similar second-order system trend due to the process’s extended dead-time scenario. Here the wired and wireless FOPPI had effective set-point tracking ability even with different performance metric results. It is worth mentioning that the offset of the wireless FOPPI controller is significantly reduced compared to the previous results. This is one of the clear indications that the FOPPI can be applied for higher-order processes even in wireless networks.

Figure 18 demonstrates the performance comparison of closed-loop control in handling packet loss conditions of wireless FOPPI controllers. Despite the wireless FOPPI controller being unable to attain the desired set-point value of two within 60 seconds, it still displayed stable control actions that maintained process stability. The sluggish response observed in this scenario can be attributed to higher system dynamics, which makes it challenging for the controller to compensate for the dead-time in the absence of the actual process variable. Nevertheless, it is noteworthy that the wireless FOPPI controller is still able to maintain stability without compromising on safety, just like the first and second-order systems. The FOPPI controller’s performance degrades beyond a 70% packet loss, resulting in an undesirable process settling with a higher offset value. Observing the controller’s actions shows that it attempts to take corrective measures to achieve the process set-point. However, the control signal’s robustness prevents it from reaching the desired value, causing the system to settle significantly below the target set-point.

## Summary and conclusions

This section presents a concise overview of the advancements of the proposed techniques in the first part. Secondly, the future research prospects and concluding remarks of this research are provided further.

### Summary

This study thoroughly analyzed the utilization of various controllers in both wired and wireless networks for a range of performance evaluation scenarios. Particular concentration is given to the dead-time compensating FOPPI controller. In order to assess the effectiveness of the controllers, simulations were conducted on several benchmark process transfer functions. The proposed FOPPI controller is specifically tested for its ability to maintain process stability under packet loss conditions. The simulation results showed that the proposed FOPPI controller demonstrated exceptional performance in settling time and peak overshoot in the wired network. Conversely, the wireless FOPPI controller proved highly effective in rise time and set-point tracking. It is essential to highlight that the findings of this study further emphasize the remarkable contributions of the proposed technique.

1. A novel FOPPI controller has been developed through the use of FOPDT and the Smith predictor algorithm, with the specific aim of addressing the extended dead-time issues that arise in both wired and wireless networks.
2. By comparing with a range of traditional controllers, including PI, FOPI, and PPI, the proposed FOPPI has demonstrated superior performance in terms of disturbance rejection, set-point tracking, and other performance metrics.
3. The proposed FOPPI controller performed well in various benchmark process models by reducing the %OS, which directly maximizes the operating lifespan of the control valve actuators.
4. While implemented in wireless networks, the FOPPI controller has successfully produced superior set-point tracking and faster rise time performance, demonstrating its strong control capability.
5. Various packet loss scenarios were simulated and analyzed on all systems to evaluate the controller’s efficacy and determine the threshold percentage of packet loss that results in system instability (i.e. 75%, 80%, and 90%).
6. In a packet loss scenario, the FOPPI controller is simulated to assess its capacity to control the process even without access to the complete process variable. The results revealed that the FOPPI could maintain process stability even with only 30% of the available process data.
7. The FOPPI controller is a simple implementation due to its analytical parameterization. However, this method requires an accurate process model in real-time applications and simulations. However, this can be solved by using a novel metaheuristic algorithm to find the controller parameters that will give better results than the traditional controller, which is currently being studied as part of ongoing research as a future scope.

### Conclusions

A fractional-order dead-time compensator has been proposed in this article to improve the controllability of wireless networks and handle longer delay compensation abilities. The FOPPI controller is simulated on benchmark first, second, and third-order systems, and the results obtained demonstrated its exceptional performance. The FOPPI controller achieved faster settling with an overall improvement of 8.3927%, and significantly reduced peak overshoot by an average of 71.4251%. As the system order increased, the FOPPI controller managed to reduce peak overshoot even further. The wireless FOPPI controller demonstrated effective tracking, quicker rise time performance, and an average improvement of 46.757% throughout the process. Moreover, FOPPI maintained process integrity and stability in wireless networks, even with only 30% of the available process data in the packet loss simulation scenario. Overall, the FOPPI controller has proven to be a highly robust and effective solution for improving the controllability of wireless networks. The performance outcome yields the ability for the controller to be applied on the wireless sensor networks to mitigate the issues of the packet loss, leading to more stable network operating conditions The effectiveness of the FOPPI controller will be validated in the future by implementing it on real-time wireless networks. Additionally, testing will be conducted under external noise conditions, and set-point and noise filters will be included in various wireless protocols.

## Data Availability

All of the data employed and created in this research has been incorporated and published within the manuscript, including its supplementary details.
